# Proteomics to study cancer immunity and improve treatment

**DOI:** 10.1007/s00281-022-00980-2

**Published:** 2023-01-04

**Authors:** Giulia Franciosa, Anders H. Kverneland, Agnete W. P. Jensen, Marco Donia, Jesper V. Olsen

**Affiliations:** 1 Novo Nordisk Foundation Center for Protein Research, University of Copenhagen, Copenhagen, Denmark; 2 National Center of Cancer Immune Therapy, Department of Oncology, Copenhagen University Hospital - Herlev and Gentofte, Herlev, Denmark

**Keywords:** Cancer immunotherapy, Tumor immunity, Proteomics, Mass-spectrometry

## Abstract

Cancer survival and progression depend on the ability of tumor cells to avoid immune recognition. Advances in the understanding of cancer immunity and tumor immune escape mechanisms enabled the development of immunotherapeutic approaches. In patients with otherwise incurable metastatic cancers, immunotherapy resulted in unprecedented response rates with the potential for durable complete responses. However, primary and acquired resistance mechanisms limit the efficacy of immunotherapy. Further therapeutic advances require a deeper understanding of the interplay between immune cells and tumors. Most high-throughput studies within the past decade focused on an omics characterization at DNA and RNA level. However, proteins are the molecular effectors of genomic information; therefore, the study of proteins provides deeper understanding of cellular functions. Recent advances in mass spectrometry (MS)-based proteomics at a system-wide scale may allow translational and clinical discoveries by enabling the analysis of understudied post-translational modifications, subcellular protein localization, cell signaling, and protein–protein interactions. In this review, we discuss the potential contribution of MS-based proteomics to preclinical and clinical research findings in the context of tumor immunity and cancer immunotherapies.

## Introduction

Over the last decade, immunotherapy has revolutionized the field of cancer treatment. Immunotherapy exploits the patient’s immune system as a natural defense against cancer. The breakthrough of immunotherapies came with the introduction of immune checkpoint inhibitors (ICIs), such as anti-CTLA4, anti-PD1, and anti-PDL1 that block inhibitory immune molecules, unleashing T cell activation [1]. The main mechanism of this treatment relies on boosting potentially tumor-reactive T cells directly in the patient’s body. As an alternative, adoptive cell therapy (ACT), utilizing either tumor-infiltrating lymphocyte (TIL)-derived T cells or T cells genetically engineered to express tumor recognizing receptors (known as CAR-T, Chimeric antigen receptor T cells), is emerging as a powerful tool [2].

Clinical efficacy of immunotherapies has been extraordinary in many cancer diagnoses, most prominently malignant melanoma, where more than half of patients obtain objective tumor regression to combination ICI therapy [3]. Still, in many patients, successful immunotherapy is hampered either due to initial lack of response (primary resistance) or development of resistance after initial response (acquired resistance) [4, 5]. Acquired resistance is emerging as a growing problem in solid tumor oncology, affecting at least 25% of patients who initially obtain a response [6].

Both primary and acquired resistance mechanisms can be attributed to tumor-intrinsic and extrinsic mechanisms. Tumor-intrinsic mechanisms are driven by the prevention of immune recognition [7]. The best example of a tumor-intrinsic mechanism of immune resistance is the expression of PD-L1, which is also targeted by ICI therapy with either anti-PD1 or PD-L1 antibodies. PD-L1 is a natural occurring checkpoint that induce T cell anergy after binding to its ligand, PD-1 [8]. Other mechanisms include downregulation of the antigen-presentation machinery and major histocompatibility complex (MHC) presentation; alterations in the interferon (IFN)-ɣ pathway; de-regulation of oncogenic signaling pathways, such as β-catenin, p53, and RAS/RAF/MAPK signaling [5]. Tumor-extrinsic mechanisms are properties in the tumor microenvironment (TME) affecting immunotherapy response. The presence of immune cells, also described as immune infiltration, is not only a good prognostic biomarker but also predictive of the effect to ICI therapy [9]. Other TME-mediated mechanisms include collagen alterations, secretion of immunosuppressive cytokines (such as IL-10 and transforming growth factor beta, TGF-β), and the depletion of essential T cell nutrition (such as tryptophan and L-arginine) [10].

Omics-based strategies have contributed in multiple ways to the fundamental understanding of anti-cancer immunity mechanisms. For instance, bulk RNA sequencing of melanoma patient samples has uncovered a primary anti-PD1 resistance (IPRES) signature [11]. Furthermore, bulk RNA sequencing of cancer cell cultures under T cell attack has revealed conserved transcriptomic changes across different cancer histological types that were shown to predict the clinical outcome after anti-PD-1/anti-PD-L1 therapy [12]. Nonetheless, transcriptomics remains limited to the detection of expressed genes that does not necessarily translate to functional differences. In addition, translational control and post-translational modifications (PTMs) can influence the gene-to-protein information process. Although initial progresses have been made [13], the study of the “translatome” in cancer immunity is still at its infancy, but in extension of genomic and transcriptomic research, advances in the proteomics field offers an even better approximation of functional changes [14]. The ability of measuring whole proteomes and the PTMs of proteins allows more accurate understanding of phenotypic cellular functions and is in the coming years likely to have a substantial impact on our understanding of anti-cancer immunity, predictive biomarkers, and drug development.

## Proteomics to study tumor immunity

Proteins are one of the main building blocks from which cells are assembled. In addition to providing the cell with shape and structure, proteins also execute nearly all its numerous functions [15]. Proteomics is the large-scale study of proteins by revealing the identity and quantity of proteins in a biological sample — cells, tissues, or body fluids.

**Fig. 1:**
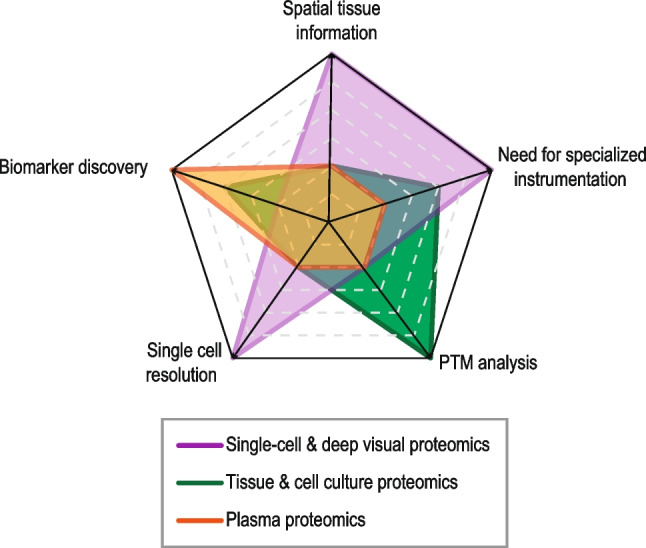
Relative comparison of three MS-based proteomics strategies that have been or can potentially be applied to the study of tumor immunity. Three different MS-based proteomics strategies are compared: single-cell and deep visual proteomics; tissue and cell culture proteomics; plasma proteomics. The comparison is based on the need of specialized instrumentation, the potential to resolve single-cell proteomes, the possibility to discover new disease biomarkers, the preservation of spatial tissue information, and the compatibility with post-translational modification (PTM) analysis. The outer the point, the better that method scores for a given trait

Given that both tumor antigens and immune checkpoints molecules are peptides and proteins, respectively, it is not surprising that mass spectrometry (MS)-based proteomics has been applied to the study of tumor immunity (Fig. 1).

**Fig. 2:**
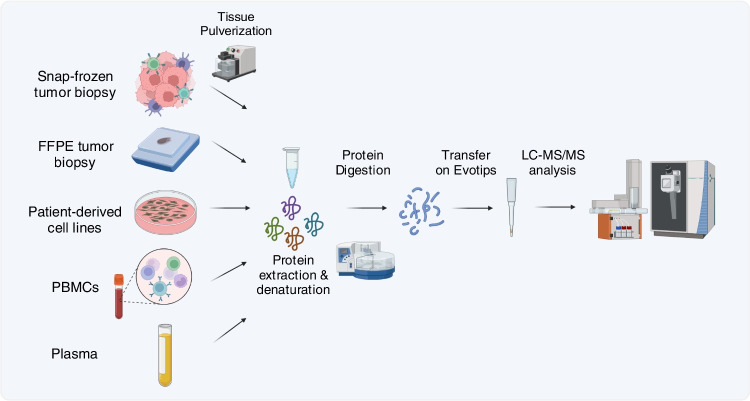
LC–MS/MS workflow for proteomics in tumor immunity studies. Proteins are extracted from snap-frozen or formalin-fixed paraffin-embedded (FFPE) tumor biopsies, patient-derived cell lines in culture, peripheral blood mononuclear cells (PBMCs), or plasma isolated from peripheral blood. For snap-frozen tissues, a preliminary pulverization step is necessary. After denaturation, proteins are digested into shorter peptides, further transferred on C18 evotips, and analyzed by liquid chromatography-tandem mass spectrometry (LC–MS/MS)

In this context, proteomics analysis can be performed on several types of biological material: tumor biopsies, TME-derived fluorescence activated cell sorting (FACS)-sorted cells, patient-derived cell cultures, peripheral blood mononuclear cells (PBMCs), plasma, and other biofluids (Fig. 2). Proteomics profiling of these sample types can help identify new protein biomarkers that can predict immunotherapy response. Furthermore, it can contribute to deciphering the properties of a certain biological system, for instance, by understanding the molecular and biological mechanisms induced by a therapeutic molecule or known extra-cellular molecules. Finally, proteomics analysis can also be used to identify new potential drug targets to be tested in pre-clinical studies.

## MS-based proteomics technology applications to cancer immunotherapy

MS-based proteomics was introduced for the first time in 1988 [16] and is now the most comprehensive approach for quantitative profiling of proteins [14], their interactions [17], and sub-cellular localization [18], and for the identification of protein PTMs [19–21].

The basic MS-based proteomics workflow, also called “shotgun” proteomics, includes the following steps. Following lysis and protein extraction from biological samples, proteins are digested into shorter peptides by cleavage with trypsin or other proteolytic enzymes. The peptide mixture is then separated through high-performance liquid chromatography (HPLC) and analyzed in the MS. The peptide mixture is transferred from the liquid phase to the gas-phase by soft ionization in an electrospray ionization (ESI) source. In ESI, the sample is sprayed out of a narrow nozzle in a high potential field, which generates multiply charged ions and almost no fragmentation [22]. The peptide ions are then analyzed in the MS by a detector, known as mass analyzer, that measures their mass-to-charge ratio (m/z) and abundance. This analysis is known as precursor or full scan (MS1 scan) and provides snapshots of peptide precursor species co-eluting at a specific time point of the LC gradient. However, the m/z ratio is not enough to univocally identify the peptide sequence. To this end, peptide ions (referred to as precursor ions) are isolated in turn and fragmented in a dedicated collision cell by collisions with inert gas molecules such as nitrogen. The obtained amino acid sequence fragments, referred to as fragment ions, are then analyzed in the mass analyzer to obtain a second spectra (MS2 or MS/MS scan). This sequential scan approach is known as MS/MS analysis or tandem MS.

For typical data-dependent acquisition (DDA) measurements, the N precursor ions of highest abundance are selected for MS/MS analysis. In the data-independent acquisition (DIA) approach, all precursor ions are sequentially isolated [23]. Consequently, each MS/MS spectrum will contain co-fragmentation information from multiple peptides, resulting in extremely complex spectra.

Finally, the sequences of peptides and associated proteins are identified by peptide search engines/algorithms, which match the precursor ion mass and its fragment ions with predicted peptide masses and their corresponding fragments derived from in silico digested protein databases [24].

### Deep proteome profiling of tumor tissue biopsies: prediction of clinical outcomes

A pioneering study from Harel et al. represented the first deep proteomic analysis of immunotherapy response [25]. The authors analyzed the proteome of 116 formalin-fixed paraffin-embedded (FFPE) tissue biopsies from stage IV melanoma patients undergoing either TIL-based or anti-PD1 immunotherapy. Bioinformatic analyses revealed higher oxidative phosphorylation and lipid metabolism in responders than non-responders in both treatments. High mitochondrial metabolism led to higher antigen presentation and IFN signaling, thereby increasing sensitivity to T cell mediated killing both in vitro and in vivo.

Proteomic analysis of FFPE tissues [26] has the advantage that samples are analyzed retrospectively, but this also bears several disadvantages. First, in FFPE tissues, PTM profiles are not fully preserved. Second, the biopsies are bulk samples which have lost all information on the complexity of the TME.

To overcome the first limitation, tissue specimens can be snap-frozen right after collection and further cryo-pulverized. This strategy would allow deep phosphoproteome profiling that can be subsequently integrated with immune profiling.

To overcome the second limitation, bioinformatic deconvolution approaches can be used to identify different cell populations within a bulk tumor. For example, xCell is a gene signature-based method that can infer up to 64 immune and stromal cell types [27]. It has been successfully used for transcriptomics data to identify four immune-based glioblastoma multiforme (GBM) subtypes [28], and could potentially be applied to deep proteome data. A similar strategy has recently been employed on proteomics data by Lehtiö et al. who applied previously described immune signatures to proteome data of 141 non-small cell lung cancer (NSCLC) tumors to evaluate which immune cell populations infiltrated the tumor site [29].

### Proteomics analysis of FACS-sorted cells and cells in culture

Another potential approach to deal with TME complexity and intra-tumor heterogeneity is the proteomic analysis of FACS-sorted cells. Multiple sample preparation protocols have been proposed. Myers et al. used a streamlined workflow that enables quantitative proteome profiling from 2 µg of protein, collected from 300,000 sorted cells per experimental condition. Utilizing a combination of facile cell collection from cell sorting, isobaric labeling for multiplexing of peptides, and small-scale fractionation, the authors profiled the proteomes of 12 freshly isolated, primary murine immune cell types [30]. Amon et al. lowered the peptide input amount to 300 ng, corresponding to 25,000 sorted cells, by using a DIA-based label-free proteomics workflow [31].

Alternatively, cells derived from TME can be cultured ex vivo before proteomic analysis. For instance, Tsai et al. successfully isolated human pancreatic primary cells and matched stromal and immune cells [32]. Andersen et al. isolated and expanded TILs from metastatic melanoma lesions and from the same lesions, they also generated primary melanoma cell lines. By co-culture assays, they were able to show that the expanded TILs recognize autologous tumors [33]. Such ex vivo experimental systems might be exploited to reduce the TME complexity and increase the protein input amount to perform deep proteomics profiling.

Primary cells could be stimulated with known cytokines or growth factors produced in the TME (e.g., IFN-γ and TNF-α). To uncover key cell signaling players, phosphoproteomics could be an interesting application approach [34]. Similar experiments can also be performed on commercial tumor cell lines. Agami’s group analyzed MD55A3 melanoma cells after IFN-γ exposure and identified numerous out-of-frame, trans-frame, and tryptophan-to-phenylalanine atypical peptides that lead to increased immune recognition. These events were induced by ribosomal frameshift following IFN-γ induced tryptophan depletion [35, 36].

Another potential proteomics application involves the study of protein–protein interactions. Purified primary murine T cells have been used to study protein–protein interactions. Celis-Gutierrez et al. performed quantitative interactomics, also known as affinity purification coupled with MS (AP-MS), to define the composition and dynamics of the PD-1 and BTLA co-inhibitory signalosomes in primary effector T cells, and at the T cell-antigen-presenting cell interface [37].

Otherwise, 2D co-cultures [33] or 3D spheroid or organoid models [38] can be established from primary cells derived from the TME. The different cell types could potentially be separated before proteomics analysis by using antibody-based magnetic separation or FACS-sorting after fluorescent cell tracer labelling.

### Immunopeptidomics for identification of targets for the T cell response

One of the most promising MS-based proteomics applications in the study of tumor immunity is the identification of tumor antigens by immunopeptidomics, which is the MS analysis of the human leukocyte antigen (HLA)-bound peptides expressed on the surface of tumor cells (extensively reviewed in [39]). The discovery of tumor antigens is essential to develop patient-tailored immunotherapies, like CAR T cell therapy and cancer vaccines.

Recognition of tumor cells by T cells requires presentation of tumor antigens on the surface of antigen-presenting cells (APCs) by HLA molecules. HLA class I (HLA-I) molecules present peptides derived mainly from proteasomal degradation of endogenous cytosolic proteins and interact with CD8 + T cells. HLA class II (HLA-II) molecules present peptides from extracellular proteins, as well as from cellular proteins degraded via the endosomal pathway, and interact with CD4 + T cells. The immunopeptidome primarily consists of peptides derived from “normal” self-proteins, with a small fraction of tumor specific peptides. Tumor antigens can be classified in canonical and non-canonical, depending if they derive from coding or non-coding regions, respectively [39]. Tumor antigens can also be classified in tumor-associated antigens (TAA), aberrantly expressed tumor-specific antigens (aeTSA), cancer-germline antigens (CGA), and mutated tumor-specific antigens (mTSA) [40]. TAAs are antigens that show superior abundance on tumor cells but are nonetheless present on normal cells and, therefore, may be subjected to central immune tolerance, leading to the inability to be recognized as “non-self” by the immune system. aeTSAs result from epigenetics-driven aberrant expression of unmutated transcripts that are not expressed in any normal somatic cell. CGAs are a sub-class of aeTSAa encoded by canonical exons normally expressed only by germ cells. mTSAs derive from mutated DNA sequences that can be either exonic or non-exonic and are believed to play a critical role in the rejection of mutated/precancerous cells by the immune system [41].

Most preclinical and clinical studies so far have employed a combination of DNA/RNA sequencing only, followed by prediction of mTSA binding based on a patient’s HLA phenotype. These methods rely on the ability of prediction algorithms to identify non-immunogenic versus immunogenic mutations, with the potential, but not experimental validation, to generate mTSA reaching the cell surface in association to HLA molecules. In contrast, pioneering studies with proteomics coupled with genetic information from Bassani-Sternberg et al. resulted in the direct identification of mTSA on the surface of cancer cells [42]. Therefore, current proteomics approaches may provide additional important information on the antigen landscape of a tumor.

To increase immunopeptidomics sensitivity, MS-based proteomics is often coupled with next-generation DNA sequencing [42], RNA sequencing (RNA-seq) [40], and ribosome-sequencing (Ribo-seq) [43] techniques. Finally, high-quality immunopeptidomics datasets can be used to improve the above-mentioned computational models for prediction of HLA-bound peptides [44].

### Plasma proteome profiling for biomarker discovery

Effective cancer immune therapy stimulates an anti-cancer immune response that is usually confined to the TME or to the organs affected by therapy toxicity. Tissue samples are inaccessible to continuous monitoring and biased by structural heterogeneity. Biological fluids are less prone to heterogeneity and provide a physiological averaging including systemic signs of activated or ongoing immune activity. Blood-based biomarkers that correlate to a successful anti-cancer immune response or other immunological outcomes, such as immune-related toxicity, would be highly desirable to fine-tune immunotherapy-based treatments to the individual patient.

Plasma is an attractive source for discovery of biomarkers due to its accessibility and relative stability. In fact, it is the preferred sample type for measuring most proteins related to host immunity including cytokines, chemokines, complement proteins, and immunoglobulins [45]. Plasma measurements are routinely used in the setting of infections, where C-reactive protein (CRP) or the precursor of calcitonin (Procalcitonin) are routinely used to monitor systemic inflammation as a surrogate of antibiotic efficacy [46]. Conversely, in the setting of ICIs, high CRP levels are generally linked to poor prognostic outcome and to immune-related toxicity [47, 48]. Individual cytokines, most notably interleukin (IL)-6 and leukemia inhibitory factor (LIF), have been correlated to clinical outcomes of therapy but can be difficult to distinguish from immune-related toxicity [49–53]. The complexity is further increased by the fact that immune-related toxicity and anti-tumor clinical efficacy are correlated outcomes [54].

To detect and monitor anti-tumor immune responses, system-level strategies are likely needed to encompass the heterogeneous dynamics and complexity of the immune system.

Plasma proteomics is the large-scale study of proteins in the plasma. Targeted protein measurement is the most common method to analyze plasma proteins. Traditionally, this has included antibody-based enzyme-linked immunosorbent assay (ELISA) or radioimmunoassay (RIA) techniques, where single proteins are measured by specific antibody binding. The methods can be multiplexed by using electrochemiluminescence or enzyme-conjugation achieving 10–20-plex assays, or even up to 500-plex when using bead-conjugated antibodies [55, 56]. Advances in next-generation sequencing (NGS) technology has also improved the ability to multiplex immunoassays. The proximity extension assay (PEA) offered by Olink can measure up 92 proteins in a panel, and it has since then been expanded to 384 proteins. If running 4 assays in parallel, this can even further increase to 1500 proteins [57, 58]. Protein-binding aptamers, offered by SomaLogic, can reportedly quantify up to 7000 proteins [59, 60]. Despite their immense potentials, targeted proteomics assays have so far not provided usable biomarkers for cancer immune therapy validated for clinical practice.

MS-based proteomics offers a different approach to plasma proteomic immune monitoring. With routine workflows (Fig. 2), around 300 proteins can be quantified in plasma whereof approximately half of the proteins are annotated to immune system processes. The main appeal of this approach is the unbiased detection and quantification of the proteins found in a plasma sample. In a recent study, plasma from 109 melanoma patients was analyzed with LC–MS/MS proteomics and identified 43 biomarker candidates including an inverse relationship of inflammation markers to favorable clinical outcomes [61]. The study identified 592 proteins across all patients but only 272 were identified across 50% of the patients. The major challenge of applying MS to analyze plasma samples is an extreme dynamic range in protein abundance. High abundant proteins (like albumin, fibrinogens, and immunoglobulins) take up 99% of the protein mass and effectively block the signal of low abundant proteins [62].

**Fig. 3:**
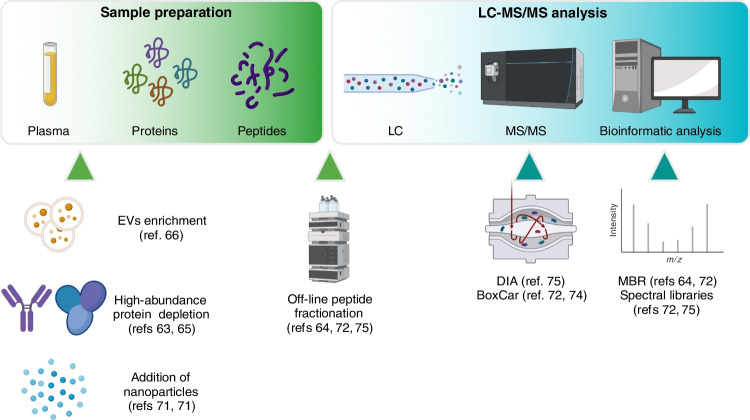
Strategies to increase sensitivity in plasma proteome profiling by MS-based proteomics. After separation of plasma from peripheral blood, several strategies can be used to increase plasma proteome depth: enrichment of extra-cellular vesicles (EVs), depletion of the most abundant proteins, or the addition of nanoparticles. Proteins are then denatured and digested into shorter peptides, which are analyzed through liquid chromatography-tandem mass spectrometry (LC–MS/MS). Data independent acquisition or BoxCar are advanced MS acquisition methods that can be used to overcome the high dynamic range of plasma samples and therefore increase plasma proteome depth. Off-line peptide fractionation can be exploited to generate deep spectral libraries to deconvolute complex DIA spectra. Alternatively, precursor information in the library can be compared to unidentified peaks in individual MS runs by using the match between runs (MBR) algorithm

Different strategies have been used to reduce the dynamic range and increase the identification of low abundant proteins in plasma (Fig. 3). Selective depletion of high abundant proteins, e.g., albumin and immunoglobulins, will increase the measurable number of proteins but will affect sample integrity, as many soluble proteins are bound to albumin [63–65]. Another approach to increase the plasma proteome depth is the enrichment of extracellular vesicles (EVs) [66]. With this strategy, detection of intracellular and membrane proteins located in the EVs are possible, providing a new biological compartment for analysis. EVs are a heterogeneous group of cell-derived membranous structures comprising exosomes and micro-vesicles that originate from the endosomal system or which are shed from the plasma membrane, respectively [67]. The main role of EVs are within intercellular communication, and they have numerous functions in immune signaling [68, 69].

The limitation of the above-mentioned strategies is the need of a relatively high amount of sample for analysis. An alternative approach is a selective enrichment of low abundant proteins by unspecific binding to nanoparticles [70]. When combining different nanoparticles with different surface chemistry, up to 2000 proteins can be quantified from plasma samples [71]. Finally, sample pooling followed by off-line fractionation techniques has been used to reduce plasma proteome complexity [72]. The obtained fractions have been used to identify MS1 spectra lacking the corresponding MS2 information through the match-between-runs algorithm implemented in MaxQuant [73]. This, combined with a special MS acquisition method known as BoxCar [74], led to the identification of more than 500 proteins per patient from only 1 µl of plasma [72]. Fractionated samples have also been used to generate spectral libraries. With this approach, reference spectra are used to deconvolute complex DIA spectra [75]. This approach led to the identification of more than 1,200 proteins in cerebrospinal fluid from 40 µl.

## Single-cell protein expression by mass cytometry

Flow cytometry is an ideal platform for evaluating the immune system at the single-cell level and is currently used to quantify labeled proteins on the surface and interior of single cells and study the immune system on the single-cell level [76]. However, conventional flow cytometers are unable to analyze the number of markers required to fully explore a single-cell proteome. This is primarily due to the limited number of fluorescently tagged markers which can be evaluated in a single tube. Instead, mass cytometry (MC) combines immunolabeling with metal-tagged antibodies together with mass spectrometry, enabling an increase in the number of markers that can be measured simultaneously up to 300–400 [77].

Withing the field of cancer immunity, two separate studies used MC to profile PBMCs from patients with melanoma before and after anti-PD-1 immunotherapy allowing the identification of pretreatment monocyte and natural killer subsets that correlated with ICI response [78, 79]. MC has also been applied to dissociated tumor tissues. To identify immune subpopulations associated with response to ICIs, Wei et al. applied mass cytometry on surgically resected melanoma tumors from patients being treated with anti-CTLA4, anti-PD1, or the combination of both, showing that anti-CTLA4 and anti-PD1-induced immune responses are driven by distinct cellular mechanisms [80]. Similarly, Gide et al. analyzed baseline melanoma dissociated tissue from patients treated with anti-PD1 monotherapy or combined immunotherapy (anti-PD1 + anti-CTLA4) identifying the subset EOMES + CD69 + CD45RO + of effector memory T cells associated with greater tumor shrinkage in both therapies [81].

In recent years, the development of commercial instrumentation that takes advantage of full spectrum fluorescence has provided the ability to increase the number of potential fluorochromes that can be effectively combined in a single panel [82]. Full spectrum flow cytometry (FSFC) can measure the entire fluorochrome emission spectrum, across multiple lasers and using many more detectors than a conventional flow cytometer, allowing a specific spectral fingerprint to be defined for each fluorochrome, allowing to combine 30–40 fluorescently labeled antibodies in a single tube. This contrasts with using a fluorochrome’s maximum emission wavelength, which defines a fluorochrome on a conventional flow cytometer. FSFC was recently benchmarked against MC and proven as an easy-to-use and high-throughput option for monitoring complex immune responses [83], which might one day replace MC in most laboratories.

The main limitation of both single-cell MC and FSFC is the inability to capture spatial information. Imaging mass cytometry (IMC) is a high dimensional tissue imaging system that allows the comprehensive and multiparametric in situ exploration of the TME at a single cell level [84]. This technique has been recently used to profile the melanoma microenvironment at various stages of disease and across different melanoma subtypes. Specifically, Moldoveanu characterized FFPE tissue from a commercially available cohort of 42 melanocytic neoplasms. Moreover, to identify TME features correlating with ICI response, they also profiled pretreatment melanoma samples from 30 patients with advanced disease who subsequently received ICI therapy (anti-PD1, anti-CTLA4 or the combination). They found that within pretreatment melanomas, the abundance of proliferating antigen-experienced cytotoxic T cells (CD8 + CD45RO + Ki67 +) and the proximity of antigen-experienced cytotoxic T cells to melanoma cells were associated with positive response to ICIs [85]. Similarly, Hoch et al. used IMC to study the chemokine landscape and immune infiltration in metastatic melanoma, highlighting major difference between “cold” and “hot” tumors [86].

## Future perspectives: towards single-cell analysis by mass spectrometry

Immunotherapy has revolutionized cancer treatment, but there are still many patients who do not obtain any benefit, or only a short-term benefit, from this type of therapy. To gain a deeper understanding of cancer immunity, new promising MS-based proteomics technologies may soon be applied to the study of the TME at the single cell level.

RNA-sequencing technologies have undergone a revolution in recent years and are now routinely applied to profile transcriptomes of single cells. High-throughput single-cell RNA-sequencing has provided important insights into cancer immunity. For example, by profiling tumor and immune cells in primary breast cancer [87], by revealing the cell type hierarchies in acute myeloid leukemia (AML) relevant for disease progression and immunity [88], and by identifying cytotoxic T cell populations associated with a positive to anti-PD1 therapy in melanoma [89]. Likewise, MS-based single-cell proteomics is now emerging as a powerful technology to study global protein expression profiles in single cells. However, contrary to genomics where DNA and RNA can be amplified by polymerase-chain reaction (PCR), MS-based proteomics is challenged by detection limitations as protein and peptide signals cannot be multiplied in a similar manner. Consequently, the MS instrumentation used to analyze single cell proteomes needs to have exquisite sensitivity to detect proteins of low cellular abundance. Proteins are typically present in copy numbers ranging from few hundred to tens of millions per cell [90]. Proteins are therefore present in about 10,000-fold higher copy numbers per cell compared to mRNAs, which typically are expressed at a few thousand copies per cell at best. This higher dynamic range of protein abundance distributions compared to mRNA transcripts also poses an additional challenge, which the MS technologies employed in single-cell proteomics must handle.

To overcome some of these challenges in single-cell proteomics, analytical strategies based on multiplexing techniques such as tandem mass tags (TMT) [91] have been devised. The isobaric TMT reagents are chemical labels that enable sample multiplexing of proteome samples by quantification and identification in tandem mass spectra. The TMT reagents have the same mass but generate reporter ions of different masses in MS/MS, which can be used for relative quantitation. Besides the possibility of analyzing up to 18 single cells in the same MS experiment, the main advantage of the TMT-based multiplexing for single-cell proteomics is possibility to boost the MS signal by an order of magnitude by introducing a carrier proteome consisting of hundreds of cells in one of the TMT channels [92]. However, although the introduction of a carrier proteome increases the overall number of protein identifications, it also dictates which proteins are identified [93] and therefore has to be used with caution. Single-cell proteomics is advancing rapidly [92, 94, 95], thanks to the continuous development of new MS instrumentation with increased sensitivity and specialized lossless sample preparation workflows. Nevertheless, protein adsorption loss during sample preparation still remains the main bottleneck for of single-cell proteomics [96], and optimized single-cell proteomics workflows therefore involves miniaturization to decrease interactions with hydrophobic surfaces, work in smallest possible volumes to keep samples as concentrated as possible and minimize buffer evaporation for sensitivity and reproducibility. Nowadays, with optimized single-cell proteomics workflows, it is possible to analyze ∼1000–3000 proteins from a single cell using label-free quantification [97] and isobaric labeling approaches [98], and up to 6,000 proteins from a few hundred cells [99].

An alternative strategy to single-cell proteomics is the recently introduced deep visual proteomics (DVP), which elegantly integrates microscopy and digital pathology with single-cell MS-based proteomics and deep learning algorithms. DVP is particularly promising for characterization of cancer tissue heterogeneity and the study of TME. It combines artificial intelligence-driven image analysis of cellular phenotypes together with automated single-cell or single-nucleus laser microdissection and high-sensitivity MS, enabling the analysis of thousands of proteins from single cell types while preserving spatial context information [100].

These novel technologies will soon be ready to become powerful tools for biomedical and translational research, including the field of cancer immunotherapy. Single-cell proteomics technologies together with the concept of deep visual proteomics is likely to revolutionize clinical proteomics and its application to study cancer immunity in the future.
